# Antibody-Based Therapeutics for Atherosclerosis and Cardiovascular Diseases

**DOI:** 10.3390/ijms22115770

**Published:** 2021-05-28

**Authors:** Eunhye Ji, Sahmin Lee

**Affiliations:** 1 Division of Cardiology, Heart Institute, Asan Medical Center, Seoul 05505, Korea; jieunhye7@gmail.com; 2 Department of Medical Science, Asan Medical Institute of Convergence Science and Technology, University of Ulsan College of Medicine, Seoul 05505, Korea

**Keywords:** atherosclerosis, inflammation, antibody therapy

## Abstract

Cardiovascular disease is the leading cause of death worldwide, and its prevalence is increasing due to the aging of societies. Atherosclerosis, a type of chronic inflammatory disease that occurs in arteries, is considered to be the main cause of cardiovascular diseases such as ischemic heart disease or stroke. In addition, the inflammatory response caused by atherosclerosis confers a significant effect on chronic inflammatory diseases such as psoriasis and rheumatic arthritis. Here, we review the mechanism of action of the main causes of atherosclerosis such as plasma LDL level and inflammation; furthermore, we review the recent findings on the preclinical and clinical effects of antibodies that reduce the LDL level and those that neutralize the cytokines involved in inflammation. The apolipoprotein B autoantibody and anti-PCSK9 antibody reduced the level of LDL and plaques in animal studies, but failed to significantly reduce carotid inflammation plaques in clinical trials. The monoclonal antibodies against PCSK9 (alirocumab, evolocumab), which are used as a treatment for hyperlipidemia, lowered cholesterol levels and the incidence of cardiovascular diseases. Antibodies that neutralize inflammatory cytokines (TNF-α, IL-1β, IL-6, IL-17, and IL-12/23) have shown promising but contradictory results and thus warrant further research.

## 1. Introduction

Cardiovascular disease is the leading cause of death in populations worldwide, and the prevalence of aging-related chronic diseases is increasing every year. According to a survey by the World Health Organization (WHO), 17.9 million people died due to cardiovascular disease in 2016, accounting for 31% of total deaths worldwide [1]. Of the cardiovascular disease-related deaths, 85% are caused by heart attacks and strokes, and the most significant cause of the two diseases is the blockage of blood vessels. Atherosclerotic lipid-laden plaques are the major etiology factors for blood vessel blockage, and as these plaques are stacked inside the sub-endothelial space (i.e., the intima), the walls of the vessel become narrow and physically interfere with the blood flow [2,3]. Since atherosclerosis is asymptomatic until the occurrence of remarkable phenomena, early detection is difficult. As such, the prognosis is poor in most cases, which directly links to the high mortality rates [2].

Yet, the currently available modes of treatment for atherosclerosis are limited to statin, ACE inhibitor, and β-blocker, among which statin is the most studied and used as first-line therapy [4]. Statin, which is used as a treatment for hyperlipidemia, functions by lowering the LDL levels; importantly, a meta-analysis of several randomized controlled studies on statin reported that statin reduced both the mortality from all causes and the incidence rates of atherosclerotic cardiovascular diseases [5]. This suggests that lipid is a critical factor in atherosclerosis.

**Figure 1:**
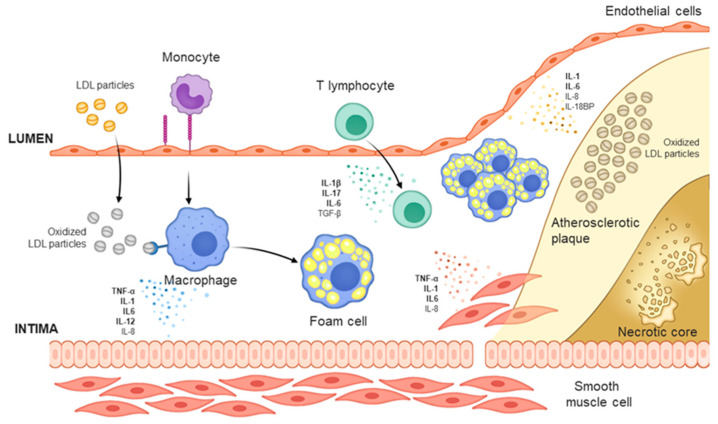
Mechanism of atherosclerosis formation.The development of atherosclerosis begins when low-density lipoprotein (LDL) particles infiltrate the intima layer and accumulate. Within the intima, LDLs form oxidized LDL (oxLDL) through myeloperoxidase and lipoxygenase, bind to the scavenger receptor of macrophage-derived foam cells, and activate the foam cells. Activated foam cells induce inflammation by secreting cytokines through several downstream signals. Concurrently, smooth muscle cells in the media layer migrate to the intima and are transdifferentiated into macrophage-like cells, and under the influence of the cytokines secreted from foam cells, secrete cytokines such as IL-6 to promote inflammation. In the intima, oxLDL increases the expression of adhesion molecules at the endothelial cell surface, leading to the recruitment of monocytes and other immune cells, and promote synergy with the aforementioned phenomena to induce the formation of atherosclerotic plaques.

Atherosclerosis has been considered to be caused by increases in cholesterol. The complexity of atherosclerosis and the involvement of various risk factors call for further research, but it is well-known that increases in cholesterol mark the beginning of atherosclerosis [6,7,8,9]. Increase in the concentration of LDL-C (low-density lipoprotein cholesterol) above the physiological need leads to the accumulation of LDL in the intima of the arteries and the development of atherosclerosis [10]. The lipid infiltrated into the intima becomes oxidized LDL (oxLDL) through oxidative modification and is engulfed by macrophages derived from monocytes to generate foam cells [11]. The form cells are held in the intima and their migration is inhibited, and thereby build up the lipid-rich center (necrotic core) of atherosclerotic plaques by being combined with cholesterol and apoptotic, necrotic cells (Figure 1) [12,13].

**Figure 2:**
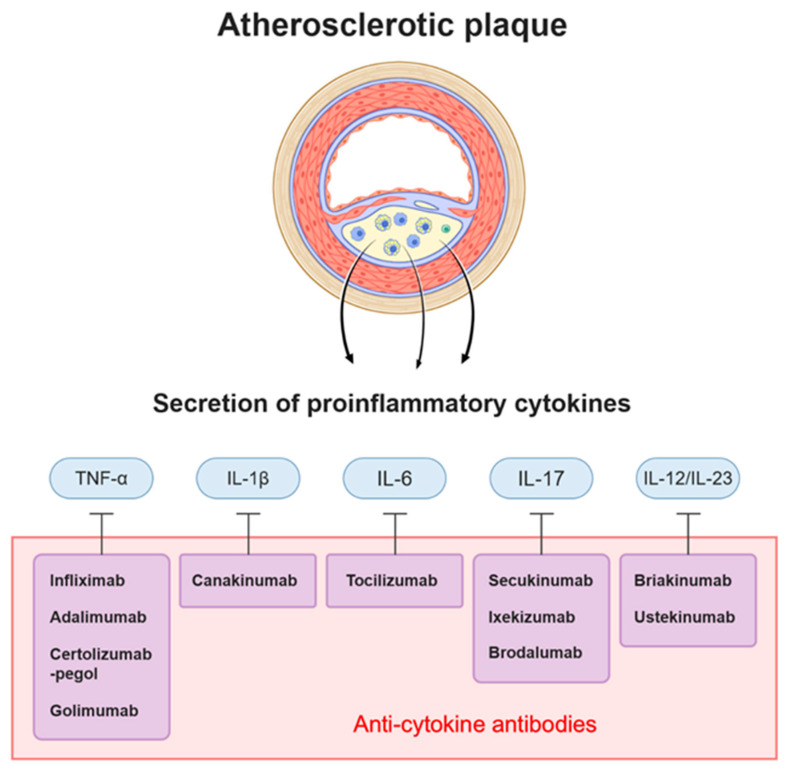
Antibodies targeting cytokines and cytokines acting on atherosclerotic plaque. Atherosclerotic plaque consists of lipid, apoptotic cells, immune cells, smooth muscle cells, and endothelial cells. These cells induce inflammation by secreting specific cytokines. Among them, IL-1β, TNF-α, IL-17, IL-6, and IL-12/23 are under investigation as therapeutic targets for atherosclerosis, and a number of antibodies have been developed to target each cytokine.

Another key mechanism that drives the development of atherosclerosis is immune/inflammation [14]. Endothelial cells at the site of the accumulation of the modified lipoprotein express VCAM-1 (vascular cell adhesion protein 1), which functions as an adhesion molecule to recruit circulating monocytes and other immune cells [15]. All cells that contribute to the development of atherosclerosis—macrophages differentiated from monocytes, recruited leukocytes, and smooth muscle cells that migrated from the media to the intima—produce and secrete various cytokines, such as tumor necrosis factor (TNF)-α, interleukin (IL)-1β, and IL-6 to promote plaque growth [16]. Through the effects of several pro-inflammatory cytokines, atherosclerosis develops and the plaques are destabilized. Accordingly, a number of antibodies have been developed to specifically target and neutralize the pro-inflammatory cytokines that are involved in the development of atherosclerotic plaques (Figure 2).

Along with the aforementioned pathogenesis studies on atherosclerosis, many pharmacological and clinical studies have been carried out [17]. This review will focus on the studies of antibody-based treatments targeting LDL and pro-inflammatory cytokines (Table 1). Therapeutic antibodies are stable molecules to be used as targeting reagents. They have an ability to bind to target proteins with high specificity and affinity. Despite of several limitations including unclear mode of action, inefficient tissue penetration and impaired immune reactions, current technological advances in antibody engineering have enabled the successful translation of antibody drugs to the clinic [18,19]. Currently, more than 79 antibody drugs are approved by the United States Food and Drug Administration (US FDA), and more than 570 antibody therapies around the world are under study [20]. Development of antibody drugs against pro-atherosclerotic factors also will play a major role in the treatment of cardiovascular diseases and inflammation.

## 2. LDL- or oxLDL-Lowering Therapies

### 2.1. Apolipoprotein B Autoantibody

Previous studies reported that high concentrations of autoantibodies that recognize various epitopes of oxidized LDL are found in atherosclerotic plaques [24,25]. In addition, animal studies using IgG antibodies specific to the epitope of oxidized LDL showed the atheroprotective effects such as decreases in atherosclerotic plaque inflammation and plaque area [26,27]. The immunization of apoE-deficient mice with MDA-p45, an MDA- modified apo B-100 peptide, increased the levels of MDA-p45 IgG and decreased atherosclerotic plaques. Experiments using anti-p45 IgG consistently demonstrated the inhibition of the development of atherosclerotic plaques, and showed the potential benefit of anti-p45 therapy [26,28]. MLDL1278a, which targets oxLDL (MDA-modified human ApoB-100), confers an anti-inflammatory effect by regulating Syk, p38 MAPK phosphorylation, and NF-κB. In subsequent experiments in obese Rhesus macaques, MLDL1278a was shown to significantly reduce pro-inflammatory cytokines and enhance the function of immune cells [29]. However, clinical results showed that the levels of MDA-modified peptide p45 and p210 autoantibody were inversely proportional to the severity of arterial disease; moreover, the GLACIER (Goal of Oxidized LDL and Activated Macrophage Inhibition by Exposure to a Recombinant Antibody) study, which was a multicenter, randomized, double-blind trial, showed that anti-oxLDL antibody did not significantly reduce carotid plaque inflammation in stable patients with cardiovascular disease [30,31,32]. In response to this trial, it was suggested that the plaque inflammation level of the patients included in the clinical trial was not high enough to be effective for the antibody therapy; however, there was no explanation for the contradiction in the results [31]. Despite some uncertainty, multiple experiments and clinical studies have demonstrated the atheroprotective effects of autoantibodies targeting oxLDL, which suggest its potential for use as an atherosclerosis antibody therapy [33].

### 2.2. PCSK9 Inhibitor

High concentration of LDL-C in the plasma is a crucial factor for atherosclerosis [34,35]. Therefore, reducing LDL-C is a key mechanism for the alleviation of the disease, and the molecular mechanism for LDL-C reduction involves PCSK9. Circulating LDL in the plasma is internalized by binding with LDL receptors on the cell surface. The internalized LDL particles are then moved to the lysosome and degraded, and the LDL receptor is recycled and expressed on the cell surface [36]. However, in the presence of PCSK9, the LDL receptor binds to PCSK9 and forms a PCSK9-LDL receptor-LDL complex. This complex is internalized into the cell and transferred to the lysosome, resulting in the degradation of both LDL and LDL receptors [37,38]. Consequentially, LDL receptors cannot be reused, and the abundance of LDL receptors on the cell surface is reduced, thereby leading to increases in the plasma level of LDL particles that serve as the trigger of atherosclerosis [37,39,40].

Accordingly, a previous study suggested that the serum level of PCSK9 may be useful as a predictive factor for early atherosclerosis, considering that the expression of PCSK9 was high in the plasma of patients with carotid IMT [41]. The E670G mutation in PCSK9 leads to an increase in enzyme activity, increases the intima-media thickness (D374Y), and decreases the level of hepatic LDL and the development of atherosclerotic plaque in pigs [42,43]. In contrast, a study using loss-of-function mutation for PCSK9 demonstrated that it was associated with the maintenance of low cholesterol levels and subsequent reduction in atherosclerotic disease [44,45]. Based on these results, PSCK9 was highlighted as the therapeutic target of atherosclerosis, and several studies have been conducted to test the potential for functional inhibition of PCSK9 using antibody mechanisms. The monoclonal antibodies that could interfere with the interaction of LDLR and PCSK9 were obtained [46,47] and it was found that these monoclonal antibodies increase the cellular LDL receptor and lower the level of LDL-C, so the clinical studies of the antibody as a PCSK9 inhibitor were promoted [48,49,50].

Two of the most well-known human monoclonal antibodies targeting PCSK9 are alirocumab (Praluent) and evolocumab (Repatha), both of which were approved in the U.S. and European Union [51]. The FOURIER (Further Cardiovascular Outcomes Research with PCSK9 Inhibition in Subjects with Elevated Risk) trial, which was conducted to verify the clinical effect of evolocumab, enrolled 27,564 high-risk patients with a history of myocardial infarction, non-hemorrhagic stroke, or symptomatic peripheral artery disease, all of whom continued to take statin while being administered with evolocumab or placebo [52,53]. The study patients were followed-up for 2.2 years and the incidence of cardiovascular abnormalities was evaluated by dividing into the primary and secondary outcomes according to gradual decreases in the LDL levels [54]. After 48 weeks, the LDL-C levels in the evolocumab group decreased by 59% compared with that in the placebo group; moreover, the incidence of the primary outcomes of MI, stroke, cardiovascular death, coronary revascularization, and unstable angina was lower in the evolocumab group (9.8%) than in the placebo group (11.3%) [34,44,52,53].

The ODYSSEY (Evaluation of Cardiovascular Outcomes After an Acute Coronary Syndrome During Treatment With Alirocumab) trial is a representative clinical trial on the safety and efficacy of alirocumab, another human monoclonal antibody targeting PCSK9. From 1315 sites, the ODYSSEY trial enrolled 18,924 patients diagnosed with acute coronary syndrome (ACS) within 12 months prior to study inclusion [55]. After 2.8 years, the LDL-c level in the alirocumab group was 54.7% lower than that in the placebo group, and the incidence of the primary outcome of non-fatal MI, fatal or non-fatal ischemic stroke, and unstable angina requiring hospitalization was lower in the alirocumab group (9.5%) than in the placebo group (11.1%) [52,56].

Unlike the evolocumab and alirocumab, the bococizumab, another antibody against PCSK9, is a humanized mouse antibody. Two large scale trials were conducted in parallel, Studies of PCSK9 Inhibition and the Reduction of Vascular Events (SPIRE) −1 and −2’, and the trials randomized 27,438 cardiovascular disease or high risk patients. The participants received 150 mg of bococizumab or placebo subcutaneously every 2 weeks. After 14 weeks, LDL-C level of bococizumab group showing 59% reduction compared with placebo. And primary endpoints were 21% lower in high-risk patients with LDL-C >100 mg/dL, but no significant results were obtained in lower-risk patients. More studies are being conducted based on these clinical results [52,57,58,59].

These clinical trials demonstrated that monoclonal antibodies against PCSK9 could dramatically lower the LDL-C level and reduce the risk of atherosclerotic cardiovascular disease. Based on these results, the 2018 ACC/AHA Multisociety guidelines recommended the use of PCSK9 inhibitors in patients with a very high risk of atherosclerotic cardiovascular disease [60,61]. In addition to the antibodies mentioned above, many studies have investigated the efficacies of LDL-lowering drugs such as PCSK9 siRNA, and bempedoic acid [53,62].

## 3. Cytokine-Targeting Therapy

### 3.1. Anti-TNF-α

TNF-α is an essential cytokine involved in adaptive immunity during the process of atherosclerosis [63]. The mRNA of TNF-α is synthesized from smooth muscle cells and macrophages present in the atherosclerotic plaques [64]. Importantly, TNF-α was steadily increased in patients after MI who were being monitored for recurrent MACE (major adverse cardiovascular events). As inflammation was shown to play a critical role in cardiovascular disease, the pro-inflammatory cytokine TNF-α was highlighted as a potent therapeutic target for cardiovascular disease [65].

In an animal experiment investigating the pathological effect of TNF-α on atherosclerosis, it was found that when the TNF-α gene was deficient, the expression levels of adhesion molecules and chemokines were altered and led to the inhibition of the development of atherosclerosis [66]. However, experiments with mice with genetic deletion of the TNF-α receptor showed contrasting results. Since TNF-α is a pro-inflammatory cytokine, it was expected that the deficiency of TNF-α would protect against atherosclerosis; however, the size of aortic atherosclerosis lesion in TNF-α-null mice was 2.3 times larger than that in wild-type mice [67].

Anti-TNF-α therapy using monoclonal antibodies that specifically bind to and neutralizes TNF-α has been studied for a long time and has led to significant advances in the treatment of rheumatoid arthritis [68]. In pilot clinical trials determining the effectiveness of anti-TNF-α monoclonal antibody on psoriatic arthritis, carotid atherosclerotic plaques were found in 15.8% of patients who received TNF-α blockers, in contrast to 40.4% of those who received traditional DMARD consisting of sulfasalazine, methotrexate, cyclosporine, and leflunomide [21]. A clinical trial using the anti-TNF-α antibody infliximab also showed that the use of TNF blocker resulted in lower events of cardiovascular disease in patients with rheumatic diseases compared with no treatment [69]. In another clinical trial that examined the 5-year cardiovascular events in patients with psoriasis, which was associated with cardiovascular disease, anti-TNF-α antibody therapy was shown to significantly reduce the cardiovascular risk compared with other treatments [70].

Aside from infliximab, other antibodies such as adalimumab, golimumab, and certolizumab pegol also target TNF-α. Adalimumab and golimumab are human monoclonal antibodies, and certolizumab pegol is a PEGylated fragment of an anti-TNF-α antibody [71]. Adalimumab therapy in psoriasis patients for 2 years significantly reduced hsCRP, E-selectin, and IL-22, and had a positive effect on reducing systemic inflammation [72]. A phase 3 clinical trial showed that compared with those who received placebo, patients with moderate-to-severe psoriasis who received certolizumab pegol were more likely to show reductions in PASI score 75 (Psoriasis Area and Severity Index score >75%) [73]. Golimumab has been approved as a monotherapy for the treatment of inflammatory arthritis such as rheumatoid arthritis and psoriatic arthritis; [74] the stability and effectiveness of golimumab were verified in several phase 3 studies, and the effect was not inferior when indirectly compared with other anti-TNF-α therapies [75].

Despite the body of experimental and clinical evidence, there are limitations in the role of anti-TNF-α therapy as its clinical benefit has not been demonstrated in heart failure. The randomized, double-blind Anti-TNF Therapy Against Congestive Heart failure study was conducted in patients with moderate-to-severe heart failure, in whom infliximab therapy for 6 weeks did not result in symptom improvement and the risk of heart failure aggravation was increased when infliximab was administered at a high dose (10 mg/kg) [76]. The mechanism of the negative outcome of high-dose anti-TNF-α therapy has not been identified. Moreover, the results in lupus, obesity, metabolic syndrome, and type 2 diabetes are inconsistent, and further studies are needed for anti-TNF-α therapy to be used in a variety of diseases [77].

### 3.2. Anti-IL-1β

IL-1 signaling leads to the expression of secondary inflammatory cytokines such as IL-6; therefore, IL-1 is a critical factor in the process of atherosclerosis [78]. The IL1 gene is translated into two forms, IL-1α and IL-1β, of which the β form plays a more important role in inflammation [79]. IL-1β exists in the inactive form, pro-IL-1β, and is cleaved by caspase 1 enzyme to be converted into the biologically active form, IL-1β [80]. The expression of IL-1β is increased through mediators such as cholesterol crystals and TNF-α [81]. IL-1β has not been studied as a biomarker for cardiovascular disease because unlike hsCRP and IL-6, it is difficult to directly measure its levels in the plasma; nevertheless, many studies have been conducted to examine its role as a therapeutic target in atherosclerosis [79].

In a murine experiment, mice with double-knockout of ApoE and IL-1β had significantly smaller sizes of atherosclerotic lesions in the aortic sinus and the ratio of atherosclerotic areas of the aorta compared with single ApoE-knockout mice [82]. On the contrary, a gain-of-function animal study on the effects of IL-1β on atherosclerosis in pigs showed that artificial expression of IL-1β on one side of the coronary artery led to increases in the coronary stenosis and aggravation of vascular diseases [83]. This result is likely due to the increase in pro-inflammatory cytokines caused by the activation of immune cells and the increase in the expression of adhesion molecules in endothelial cells [84,85]. The results of these animal experiments served as the basis for clinical trials on anti-IL-1β therapy.

Canakinumab, a human monoclonal antibody for IL-1β, has been approved for use in a variety of rheumatic inflammatory diseases including cryopyrin-associated periodic syndrome, systemic juvenile idiopathic arthritis, and adult-onset Still’s disease [86]. In addition, studies have shown that canakinumab significantly reduces inflammation, regardless of LDL-C or HDH-C, which suggests that canakinumab can be used as a therapeutic agent that inhibits the inflammatory response of atherosclerosis [87].

The Canakinumab Anti-Inflammatory Thrombosis Outcome Study (CANTOS) was carried out by enrolling 17,200 patients with coronary artery disease after MI whose high-sensitivity C-reactive protein (hsCRP) level steadily increased and remained at high cardiovascular risk despite secondary prevention medical therapy such as statin [88]. The patients were administered either placebo or canakinumab (50, 150, or 300 mg) for three months, and examination at 48-months showed that the hsCRP levels in the canakinumab 50 mg group, 150 mg group, and 300 mg group were 26%, 37%, and 41% of that of the placebo group [89]. At an intermediate follow-up of 44 months, the incidence of the primary endpoints was lower in the canakinumab-treated groups (50 mg: 4.11/100, 100 mg: 3.86/100, 300 mg: 3.9/100) compared with that of the placebo group (4.5/100) [89]. In conclusion, CANTOS showed that canakinumab can reduce the risk of cardiovascular disease by lowering inflammation without altering the lipids [77,90]

### 3.3. Anti-IL-6

IL-6, which is induced by pro-inflammatory cytokines such as IL-1 and TNF cytokines, acts as a central hub for atherosclerosis inflammatory signaling. IL-6 is produced in various cells such as smooth muscle cells, endothelial cells, and immune cells, and is a soluble cytokine that can move away from the source of inflammation and reach a target tissue by blood circulation [91]. In a study investigating the association of calcified coronary atherosclerosis and IL-6 in patients with type 2 diabetes, the level of IL-6 identified a significant association with the coronary arterial calcium score independent of other cardiovascular risk factors [92]. This study demonstrated the potential use of IL-6 as a risk factor for coronary atherosclerosis and as a therapeutic target for atherosclerosis.

One study examined the effects of IL-6 on the development of early atherosclerosis in non-obese diabetic male mice and ApoE-deficient mice that were fed a high-fat or normal chow diet for 15 weeks while receiving recombinant IL-6 or saline once a week. Regardless of the genetic alteration and diet, all mice treated with recombinant IL-6 showed significant increases in the levels of the pro-inflammatory cytokines IL-1β, TNF-α, and fibrinogen; more importantly, the area of fatty streak lesion were 1.9- to 5.1- fold larger compared with that in saline-treated mice [93]. However, IL-6 has a two-sided role in the development of atherosclerosis. In mice with double-knockout of ApoE and IL-6, serum cholesterol concentration and atherosclerotic lesion area were significantly higher compared with ApoE single-knockout mice [94]. Another study using young IL-6 and ApoE double-knockout mice showed no significant differences in the development of fatty streaks in comparison with mice of other genotypes (IL-6^+/+^ApoE^−/−^, IL-6^+/−^ApoE^−/−^) [95]. IL-6 is generally recognized as a pro-inflammatory cytokine, but it also plays a role in lowering pro-inflammatory activity by releasing soluble TNF receptors; therefore, the balance of IL-6 activity is critical in the pathogenesis of atherosclerosis [16].

IL-6 is also expressed in human atherosclerotic plaque, and an investigation of the correlation between IL-6 and risk factors of cardiovascular disease in healthy individuals showed that high IL-6 levels were associated with the risk of atherosclerosis-associated MI [96]. In addition, the variant (rs7529229) of the IL-6 receptor, which increases the level of circulating IL-6 and lowers the concentration of C-reactive protein and fibrinogen, reduced the risk of coronary heart disease events [97]. As a result of several studies commonly showing the relationship between IL-6 and atherosclerosis, IL-6 became a therapeutic target for atherosclerosis [68].

Tocilizumab, a humanized monoclonal antibody for the IL-6 receptor, interferes with the binding between IL-6 and IL-6 receptors and has been approved as a treatment in RA [63]. Tocilizumab raises the level of LDL-cholesterol, triglycerides, and HDL-cholesterol to deteriorate the lipid profile; [98] however, several studies have also reported positive results. In cohort studies using claims data from Medicare, IMS PharMetrics, and MarketScan, tocilizumab was not associated with an increased risk of cardiovascular while being associated with protective effects against MACE outbreaks [99]. Similar studies have been published in Italy [100] and Japan as well [101]. Overall, tocilizumab increases the plasma levels of LDL-C and triglyceride, but reduces the incidence of MACE. The ongoing Assessing the Effect of Anti-IL-6 Treatment in Myocardial Infraction (ASSIL-MI) phase II trial will evaluate whether tocilizumab can reduce myocardial damage in patients with ACS [102].

### 3.4. Anti-IL-17

IL-17 is divided into 6 members from A to F, of which A and F are the most critical members. IL-17 is produced from immune cells, such as CD4+ T helper cells, Tc17 cells, natural killer cells, and natural killer T cells [103]. IL-17a plays a role in the protection against bacterial or fungal infection, and it was found that the sensitivity to infection increases in cases with defects in IL-17A or malfunctions in the IL-17 receptor [104]. In addition, IL-17 has shown therapeutic benefits in chronic inflammatory disorders such as psoriasis, rheumatoid arthritis, and inflammatory bowel disease, a type of Crohn’s disease [105,106].

The mechanism of IL-17 for atherosclerosis remains under debate. Several studies have shown that IL-17A increases the formation of atherosclerotic plaques [107,108,109], but not others [110,111]. The ambivalence of the role of IL-17A may be due to differences in the surrounding environment such as the location of the plaque and the level of other cytokines and chemokines [112]. Other studies reported the protective effect of IL-17A by reducing endothelial expression of VCAM-1 used for monocyte adhesion and stabilizing atherosclerotic plaque [111,113,114]. In addition, in a cohort study of more than 1000 patients with acute MI, low IL-17 levels were associated with the relapse of major adverse cardiovascular events within one year after cardiovascular risk factor treatment [115].

Secukinumab is the first human monoclonal antibody among antibody drugs targeting IL-17 that has been approved for clinical use, and has shown positive effects in psoriasis, psoriatic arthritis, and ankylosing spondylitis [22,116,117,118,119]. Secukinumab has shown significantly better effects than Ethanercept, a receptor that targets TNF-α [116,120], and adalimumab, an antibody that targets TNF-α to offset its functionality [121]. In addition, long-term use of secukinumab was associated with only low-risk side effects such as cold and diarrhea [103]. Another monoclonal antibody targeting IL-17A is lxekizumab, which was recently approved for use in plaque psoriasis. In a clinical trial conducted on patients with plaque psoriasis, the plaque was completely cured in 34% to 37% of all patients [122]. Brodalumab acts on the A chain of the IL-17 receptor and blocks the binding of IL-17 and the receptor, thereby interfering with the downstream signaling pathway. Compared to secukinumab, brodalumab showed a better effect on psoriasis [123]. However, IL-17 neutralization studies conducted on patients with rheumatoid arthritis did not report consistent results [124,125]. Therefore, it is necessary to further study the mechanism of functional changes in IL-17 in various situations and environments and to discover therapeutic candidates to offset the function of IL-17.

### 3.5. Anti-IL-12/23

IL-23 and IL-12 are cytokines constituting the same IL-12 family and act as heterodimers, each of which shares IL-12p40 and forms a dimer with IL-23p19 and IL-12p35. These two cytokines are produced by immune cells such as macrophages and bind to respective receptors to activate the JAK-STAT signaling and regulate inflammation [126,127]. High levels of serum IL-12 were detected in animal experiments using atherosclerosis-induced ApoE knockout mice [128]. Treatment with recombinant murine IL-12 in ApoE knockout mice and LDLR-deficient mice significantly increased the aortic atherosclerotic plaque areas [129]. Conversely, when the function of IL-12 was neutralized using vaccination in LDL receptor-knockout mice, atherogenesis was reduced by 68.5% [130,131]. The levels of IL-12 family cytokines are also significantly higher in patients with atherosclerosis associated with cardiovascular disease [132,133].

Since IL-12 and IL-23 share the IL-12p40 subunit, briakinumab and ustekinumab targeting IL-12p40 are able to neutralize both IL-12 and IL-23. Briakinumab is a human monoclonal antibody, and its efficacy was tested in a small-sized preclinical study involving patients with moderate-to-severe Crohn’s disease who received either placebo or briakinumab (1 or 3 mg/kg) for 7 weeks [134]. In the initial treatment results, the 3 mg/kg injection group showed a significantly higher response rate compared with the 1 mg/kg injection and the placebo group, but the statistical significance of the difference disappeared during the 18-week follow-up period [134]. In addition, several meta-analyses compared antibody-based inflammatory agents with briakinumab and showed that MACE was more common in the briakinumab-treated groups [23].

Another monoclonal antibody targeting the human IL-12p40 is ustekinumab, which has been recently approved for use in Crohn’s disease, following its approval for psoriasis and psoriatic arthritis [126]. Ustekinumab was found to be effective in a phase 2a study conducted in patients with moderate-to-severe Crohn’s disease, and a randomized, double-blind phase 2b study showed an increased response rate in patients with moderate-to-severe Crohn’s disease in whom anti-TNF therapy was not effective [135]. The subsequent phase 3 trial was designed more precisely and showed that the 6-week induction trial had significantly higher responses rates in the ustekinumab group than in the placebo group, and some of the patients who received ustekinumab in the induction trial were enrolled in the main maintenance study [136]. The main study measured the rate of remission of the disease at week 44, and the rate of remission was higher in the ustekinumab group than in the placebo group (placebo: 35.9%, ustekinumab every 8 weeks: 53.1%, ustekinumab: 48.8%). These results demonstrated the effectiveness of ustekinumab in alleviating the symptoms of patients with moderate-to-severe Crohn’s disease [136]. A small-scale pilot study examined the atherosclerosis parameters in patients with psoriasis treated with ustekinumab, and reported that while there was a noticeable relief of skin lesions after 6 months of treatment, there was no notable change in the pulse wave velocity and intima-media thickness [137].

## 4. Discussion

This review summarized the mechanisms of action of antibody-based treatments targeting LDL and cytokines, which are the major causes of cardiovascular disease and atherosclerosis, and their results in recent clinical trials. The results of antibody therapy are ambivalent, with some cases showing significant alleviation of symptoms and others experiencing adverse events such as the aggravation of cardiovascular diseases. Antibodies targeting IL-17a and IL-12/23 also acted as pathogens in some cases, and briakinumab was withdrawn from the market due to increases in MACE. Therefore, it is important to monitor the side effects of new antibody therapies in terms of cardiovascular disease. Delineating the exact mechanism of action of the target molecules would be very helpful in overcoming the side effects or applying the appropriate treatment according to the situation and environment of each patient. In addition to the antibodies mentioned in the text, development and clinical trials of antibodies that inhibit a variety of molecules continue, such as ANGPTL family, CD47, CD31 [138,139,140,141]. Through these efforts, more targets will be found in the future, and mediators such as specific antibodies will be developed and eventually lead to the conquering of many diseases.

## Figures and Tables

**Table 1:** Summary of antibody based clinical trials.

Therapeutic/Study Name	Antibody Name	Target	Patients	Result
GLACIER	MLDL1278A	oxLDL (MDA-modified human ApoB-100)	CVD patients	Non significantly reduce carotid plaque
FOURIER	Evolocumab	PCSK9	patients with clinically evident CVD(prior MI, stroke or PAD)	LDL-C level and primary outcomes (MI, stroke, cardiovascular death, coronary revascularization, unstable angina) reduction
ODYSSEY	Alirocumab	PCSK9	patients diagnosed with ACS	LDL-C level and primary outcomes (non-fatal MI, ischemic stroke, unstable angina) reduction
SPIRE	Bococizumab	PCSK9	CV or high risk patients	LDL-C level and primary ennpoint reduction in LDL-C >100 mg/dL group
ATTACH	Infliximab	TNF-α	Heart failure	Deteriorated heart failure
STROBE (follow up study)	Infliximab	TNF-α	Psoriasis	Significantly reduce the cardiovascular risk
Di Minno et al. [21]	Adalimumab, Infliximab	TNF-A	Psoriatic arthritis	Decreased atherosclerosis of carotid artery
CANTOS	Canakinumab	IL-1B	CAD after MI + hsCRP	Decreased hsCRP level and incidence of the primary endpoint (nonfatal myocardial infarction, stroke, cardiovascular death)
ASSIL-MI	Tocilizumab	IL-6	ACS	Increased myocardial salvage
Mease et al. [22]	Secukinumab	IL-17	Psoriatic arthritis	Non significant increased MACE
Uncover	Ixekizumab	IL-17	Moderate to severe psoriasis	Reduced Psoriasis Area and Severity Index (PASI) score
Langley et al. [23]	Briakinumab	IL-12/23	Psoriasis	Increased MACE
Uniti	Ustekinumab	IL-12/23	Moderate to severe Crohn’s disease	Significantly higher rate of response
